# Calcium-Dependent Protein Kinase 5 Is Required for Release of Egress-Specific Organelles in *Plasmodium falciparum*

**DOI:** 10.1128/mBio.00130-18

**Published:** 2018-02-27

**Authors:** Sabrina Absalon, Karin Blomqvist, Rachel M. Rudlaff, Travis J. DeLano, Michael P. Pollastri, Jeffrey D. Dvorin

**Affiliations:** a Division of Infectious Diseases, Boston Children’s Hospital, Boston, Massachusetts, USA; b Department of Pediatrics, Harvard Medical School, Boston, Massachusetts, USA; c Department of Microbiology, Tumor and Cell Biology, Karolinska Institutet, Stockholm, Sweden; d Biological and Biomedical Sciences, Harvard Medical School, Boston, Massachusetts, USA; e Department of Chemistry and Chemical Biology, Northeastern University, Boston, Massachusetts, USA; NIAID, NIH

**Keywords:** *Plasmodium falciparum*, calcium-dependent protein kinase, egress, malaria, microneme

## Abstract

The human malaria parasite *Plasmodium falciparum* requires efficient egress out of an infected red blood cell for pathogenesis. This egress event is highly coordinated and is mediated by several signaling proteins, including the plant-like *P*. *falciparum* calcium-dependent protein kinase 5 (PfCDPK5). Knockdown of PfCDPK5 results in an egress block where parasites are trapped inside their host cells. The mechanism of this PfCDPK5-dependent block, however, remains unknown. Here, we show that PfCDPK5 colocalizes with a specialized set of parasite organelles known as micronemes and is required for their discharge, implicating failure of this step as the cause of the egress defect in PfCDPK5-deficient parasites. Furthermore, we show that PfCDPK5 cooperates with the *P*. *falciparum* cGMP-dependent kinase (PfPKG) to fully activate the protease cascade critical for parasite egress. The PfCDPK5-dependent arrest can be overcome by hyperactivation of PfPKG or by physical disruption of the arrested parasite, and we show that both treatments facilitate the release of the micronemes required for egress. Our results define the molecular mechanism of PfCDPK5 function and elucidate the complex signaling pathway of parasite egress.

## IMPORTANCE

The signs and symptoms of clinical malaria result from the replication of parasites in human blood. Efficient egress of the malaria parasite *Plasmodium falciparum* out of an infected red blood cell is critical for pathogenesis. The *P*. *falciparum* calcium-dependent protein kinase 5 (PfCDPK5) is essential for parasite egress. Following PfCDPK5 knockdown, parasites remain trapped inside their host cell and do not egress, but the mechanism for this block remains unknown. We show that PfCDPK5 colocalizes with parasite organelles known as micronemes. We demonstrate that PfCDPK5 is critical for the discharge of these micronemes and that failure of this step is the molecular mechanism of the parasite egress arrest. We also show that hyperactivation of the cGMP-dependent kinase PKG can overcome this arrest. Our data suggest that small molecules that inhibit the egress signaling pathway could be effective antimalarial therapeutics.

## INTRODUCTION

Malaria remains an important cause of global morbidity and mortality with an estimated 214 million cases and 438,000 deaths in 2015 (1). Most of these deaths are due to infection by the *Plasmodium falciparum* parasite. The emergence and spread of drug resistance, particularly of artemisinin-resistant parasites in southeastern Asia (2), threaten to limit the usefulness of antimalarial therapies. Therefore, there is an ongoing need to identify additional critical biological pathways that might be targeted by a new generation of antimalarial medications.

The major clinical manifestations of malaria result from exponential expansion of parasites during the blood stage of the life cycle (3–5). Asexual blood-stage development of *Plasmodium* parasites occurs via schizogony. In this process, multiple asynchronous nuclear divisions occur within a common cytoplasm. Mature and invasive merozoites are not formed until a final segmentation (or budding) wherein the newly formed daughter parasites are all formed simultaneously. Thus, the daughter merozoites are not fully mature until all daughter merozoites are mature (6). This requirement highlights the importance of a coordinated egress signaling pathway to prevent release of immature parasites.

In apicomplexan organisms, including both *Plasmodium* spp. and *Toxoplasma gondii*, release of specialized apical organelles known as micronemes is a critical step in parasite egress. The triggered discharge of these organelles depends on the activation of calcium signaling, cGMP signaling, and phosphatidic acid signaling (7–10). Some of the protein mediators of microneme discharge have been identified, including calcium-dependent protein kinases (7, 11), C2 domain-containing proteins (12), and acylated pleckstrin homology domain-containing proteins (10). However, the specific roles played by these proteins in the activation of microneme discharge remain incompletely understood.

Calcium-dependent protein kinases (CDPKs) (13–15) and the cyclic GMP-dependent protein kinase (PKG) (8, 16, 17) are important for *P. falciparum* egress. Additionally, a protease cascade mainly driven by the serine protease *P. falciparum* SUB1 (PfSUB1) is essential for egress (8, 18, 19). *P. falciparum* PKG (PfPKG) activity is required for PfSUB1 release from specialized exoneme organelles (8), and once discharged into the parasitophorous vacuole, PfSUB1 proteolytically processes multiple parasite proteins, including the *P. falciparum* merozoite surface protein-1 (PfMSP1), which itself is essential for egress and allows PfMSP1 to interact with and destabilize the host erythrocyte cytoskeleton (20). An important role for the perforin-like protein PfPLP1 in blood-stage parasite egress has been hypothesized (21). However, PfPLP1 can be knocked out in the blood stage without a detectable replication defect, suggesting that its role is nonessential and/or redundant for asexual parasite egress (22). PfCDPK5 (PF3D7_1337800) is essential for *P. falciparum* egress (15). However, the mechanism behind the egress block in PfCDPK5-deficient parasites has remained unknown.

In the current study, we perform a functional evaluation of the egress defect in PfCDPK5-deficient parasites. With superresolution microscopy, we demonstrate the dynamic localization of PfCDPK5 throughout schizogony and its colocalization with a subset of microneme organelles. We show that PfCDPK5-deficient parasites do not efficiently release a subset of micronemes and that this failure of microneme release is the cause of the egress block. In response to baseline activation of PfPKG, PfCDPK5-deficient parasites initially show activation of the protease cascade that is similar to parasites without PfCDPK5 deficiency. However, this level of PfPKG activation is insufficient to induce the release of the required subset of micronemes for egress, and protease processing does not efficiently proceed to completion in PfCDPK5-deficient schizonts. Pharmacological enhancement of PfPKG activation facilitates microneme release and overcomes the PfCDPK5-mediated deficiency. Interestingly, mechanical release of PfCDPK5-deficient merozoites also overcomes this block. We demonstrate that a major function of PfCDPK5 is to act cooperatively with the PfPKG signaling pathway. Our study provides a new layer in our understanding of the *P. falciparum* egress pathway.

## RESULTS

### PfCDPK5 has dynamic localization during schizont development.

**FIG 1:**
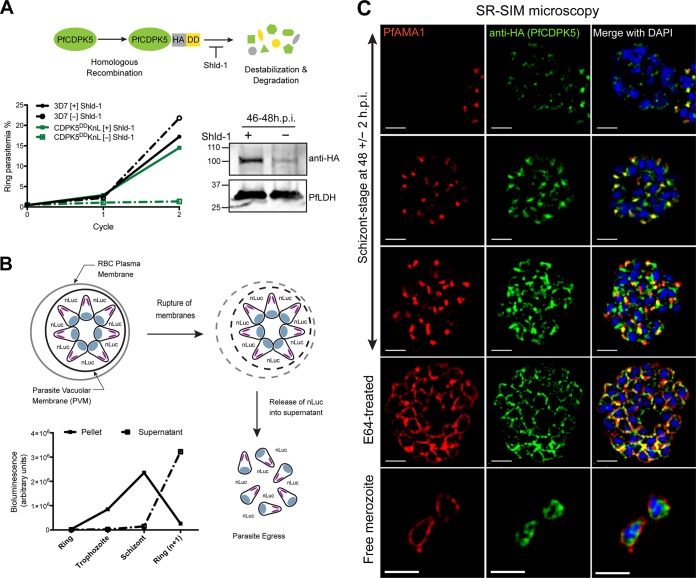
Characterization of 3D7-PfCDPK5^DD^KnL parasites and PfCDPK5 localization. (A, top) Schematic of 3D7-PfCDPK5^DD^KnL regulation. This parasite strain has two genetic modifications. The destabilizing domain (DD) is fused to the carboxy terminus of PfCDPK5, and the KnL reporter gene is integrated into the genome. The PfCDPK5 fusion protein is stabilized in the presence of Shld-1 and degraded in the absence of Shld-1. (Bottom left) Replication curves of 3D7-PfCDPK5^DD^KnL and control 3D7 parasites cultured with Shld-1 ([+] Shld-1) and without Shld-1 ([−] Shld-1). Values are means ± standard deviations (SD) (error bars) (*n* = 3). (Bottom right) Immunoblot of schizont-stage lysates probed with anti-HA (recognizes the epitope tag of PfCDPK5-3HA-DD) and anti-PfLDH (loading control). The positions of molecular mass markers (in kilodaltons) are indicated to the left of the blot. h.p.i., hours post-invasion. (B) Schematic of KnL reporter protein release from 3D7-PfCDPK5^DD^KnL parasites. The graph shows bioluminescent activity in supernatant and parasite pellets from ring, trophozoite, schizont, and reinvaded parasites. Values are means ± SD (*n* = 3). (C) Schizonts from [+] Shld-1 3D7-PfCDPK5^DD^KnL parasites were fixed, probed with anti-HA and anti-PfAMA1 antibodies, and visualized by SR-SIM. Bars, 2 µm.

We generated a new transgenic *P. falciparum* strain, 3D7-PfCDPK5^DD^KnL, with three copies of the hemagglutinin (HA) epitope followed by the destabilizing domain (DD) at the endogenous PF3D7_1337800 locus (Fig. 1A; also see Fig. S1 in the supplemental material). By placing the DD at the carboxy terminus of *P*. *falciparum* calcium-dependent protein kinase 5 (PfCDPK5), the resulting fusion protein is rapidly degraded when the stabilizing agent Shield-1 (Shld-1) is removed. As expected, when grown in the absence of Shld-1, these parasites have a >90% schizont-stage replication defect in the second cycle (Fig. 1A). These parasites also have a single copy of the mycobacteriophage Bxb1 *attB* sequence (23). To allow quantitative measurement of egress following parasitophorous vacuolar membrane (PVM) and red blood cell (RBC) plasma membrane rupture, we integrated an *attP*-containing nano-luciferase reporter cassette into this *attB* site (24, 25). By fusing the first 60 amino acids from the knob-associated histidine-rich protein, the nano-luciferase reporter protein (referred to as KnL) is targeted via the secretory system to the parasitophorous vacuole (26). In parasite cultures grown with Shld-1 ([+] Shld-1), bioluminescence accumulates in the cell pellet during asexual development and is released into the supernatant following egress (Fig. 1B). Thus, parasite biomass can be quantified by bioluminescence in cultured cell pellet, and egress is monitored by release of the KnL into the cell-free supernatant.

10.1128/mBio.00130-18.2 FIG S1  Generation of transgenic parasites. (A) Schematic of single-crossover vector (pJDD253) used to generate 3D7-PfCDPK5^DD^ parasites with the *attB* site and integration vector (pJDD250) to add KnL reporter gene via the *attP* site to generate 3D7-PfCDPK5^DD^KnL parasites. (B) PCR verification of integration. Primer pair a amplifies the internal region of PfCDPK5 and generates a product in the parental 3D7, the clone of 3D7-PfCDPK5^DD^, and the 3D7-PfCDPK5^DD^KnL parasites. Primer pair b generates a product only after pJDD253 integration in 3D7-PfCDPK5^DD^ and its daughter 3D7-PfCDPK5^DD^KnL parasites. Finally, primer pair c generates a product only after pJDD250 integration in the 3D7-PfCDPK5^DD^KnL parasites. We note that there is a minor and smaller background band with primer pair c in the 3D7-PfCDPK5^DD^ clone. This background band is also seen below the correct larger band with primer pair c in 3D7-PfCDPK5^DD^KnL parasites. Download FIG S1, TIF file, 22 MB. Copyright © 2018 Absalon et al. 2018 Absalon et al. This content is distributed under the terms of the Creative Commons Attribution 4.0 International license.

To evaluate the localization of PfCDPK5 during the asexual life cycle of *P. falciparum*, we utilized superresolution structured illumination microscopy (SR-SIM). Parasites were tightly synchronized, and at 44 h post-invasion (h.p.i.), schizonts were treated with E64, a cysteine protease inhibitor that prevents egress but still allows maturation of viable merozoites (26–28). Samples were collected for immunofluorescence analysis (IFA) by SR-SIM at ~48 ± 2 h.p.i. For an internal “timer” for parasite maturation, we monitored the expression and localization of the *P. falciparum* apical membrane antigen-1 (PfAMA1) (29–31). Consistent with previous studies of PfAMA1, we initially detected PfAMA1 expression in the micronemes and after a poorly characterized “egress trigger,” observed PfAMA1 translocation to the plasma membrane of the fully mature daughter merozoites (8, 32, 33). In 3D7-PfCDPK5^DD^KnL parasites with undetected PfAMA1 expression, we observed punctate cytoplasmic localization of PfCDPK5 (Fig. 1C, top row). In parasites with detected micronemal PfAMA1 localization, we observed two different staining patterns for PfCDPK5. The first was observed in parasites with weaker PfCDPK5 staining, where there was strong colocalization with micronemal PfAMA1 (Fig. 1C, second row). Given that PfCDPK5 has no known signal sequence, we hypothesize that it is located on the cytoplasmic side of these micronemes. In the second pattern, observed at higher levels of PfCDPK5 expression, PfCDPK5 retained colocalization with PfAMA1 but also exhibited additional diffuse apical staining (Fig. 1C, third row). In parasites that were beyond the “egress trigger,” PfAMA1 was translocated to the merozoite surface, and PfCDPK5 relocalized to a peripheral cytoplasmic distribution with increased signal near the plasma membrane (Fig. 1C, E64-treated row). Localization was similar in free merozoites isolated from parallel cultures grown without E64 (Fig. 1C, Free merozoite row). Complete z-stacks for a representative parasite from each localization type are provided (see Movies S1 to S5 in the supplemental material). To verify that PfCDPK5 was localized inside the cytoplasm of “post-egress trigger” merozoites, we costained the parasites with antibodies directed against the PfGAP45 protein associated with the inner membrane complex (34) and confirmed that PfCDPK5 staining is located within PfGAP45 staining (Fig. S2A). This result demonstrates that PfCDPK5 is located within the parasite cytoplasm.

10.1128/mBio.00130-18.3 FIG S2  (A) SR-SIM IFA of 3D7-PfCDPK5^DD^KnL schizont stained with antibodies against HA epitope (green) and PfGAP45 (red). Parasite nuclei were counterstained with DAPI (blue). The side and top panel show reconstructed views in perpendicular planes. The PfCDPK5 signal is inside the merozoites. (B) SR-SIM IFAs of 3D7-PfCDPK5^DD^KnL schizonts stained with antibodies against HA epitope (green) and PfSUB1 or PfRON4 (red). While there is some overlap with the diffuse apical staining of PfCDPK5, strong colocalization is not demonstrated for these apical markers. Download FIG S2, TIF file, 14.8 MB. Copyright © 2018 Absalon et al. 2018 Absalon et al. This content is distributed under the terms of the Creative Commons Attribution 4.0 International license.

10.1128/mBio.00130-18.6 MOVIE S1  Schizont prior to expression of PfAMA1. The supplemental movies show serial z-sections to evaluate localization of PfCDPK5 (anti-HA [green]) and PfAMA1 (anti-PfAMA1 [red]). The parasite nuclei are counterstained with DAPI (blue). The timing of the five schizonts is determined by the localization and amount of PfAMA1 staining. Download MOVIE S1, MOV file, 0.6 MB. Copyright © 2018 Absalon et al. 2018 Absalon et al. This content is distributed under the terms of the Creative Commons Attribution 4.0 International license.

10.1128/mBio.00130-18.7 MOVIE S2  Schizont expressing low levels of micronemal PfAMA1. See the legend to Movie S1. Download MOVIE S2, MOV file, 1 MB. Copyright © 2018 Absalon et al. 2018 Absalon et al. This content is distributed under the terms of the Creative Commons Attribution 4.0 International license.

10.1128/mBio.00130-18.8 MOVIE S3  Schizont expressing relatively higher levels of micronemal PfAMA1. See the legend to Movie S1. Download MOVIE S3, MOV file, 0.8 MB. Copyright © 2018 Absalon et al. 2018 Absalon et al. This content is distributed under the terms of the Creative Commons Attribution 4.0 International license.

10.1128/mBio.00130-18.9 MOVIE S4  E64-treated schizont with translocated PfAMA1. See the legend to Movie S1. Download MOVIE S4, MOV file, 0.8 MB. Copyright © 2018 Absalon et al. 2018 Absalon et al. This content is distributed under the terms of the Creative Commons Attribution 4.0 International license.

10.1128/mBio.00130-18.10 MOVIE S5  Free merozoite with plasma membrane-localized PfAMA1. See the legend to Movie S1. Download MOVIE S5, MOV file, 0.2 MB. Copyright © 2018 Absalon et al. 2018 Absalon et al. This content is distributed under the terms of the Creative Commons Attribution 4.0 International license.

**FIG 2:**
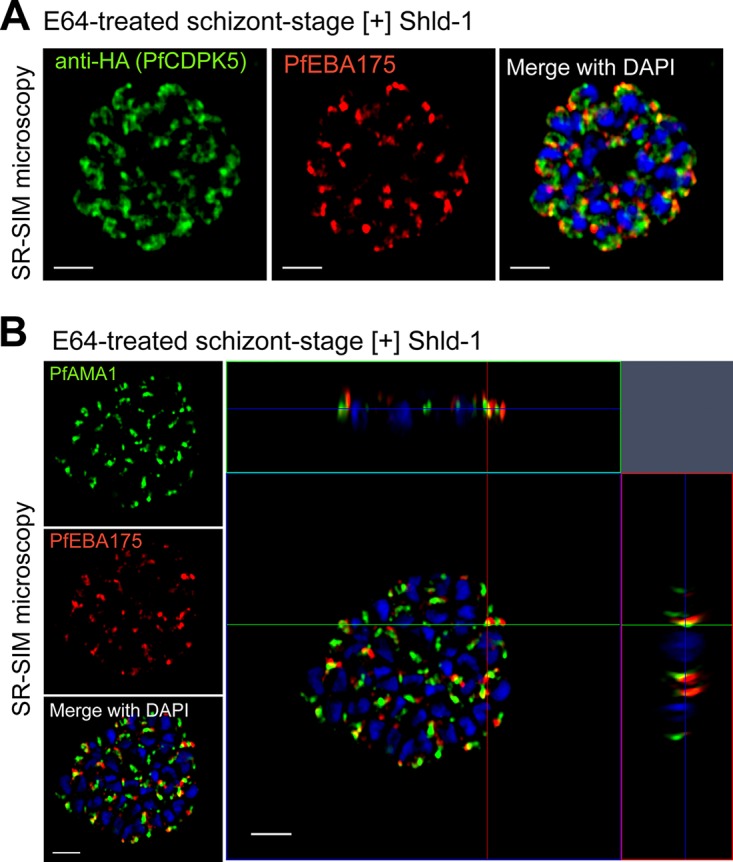
Localization of PfEBA175, PfAMA1, and PfCDPK5. (A and B) Schizonts from [+] Shld-1 3D7-PfCDPK5^DD^KnL parasites were fixed, probed with anti-HA and anti-PfEBA175 (A) or anti-PfAMA1 and anti-PfEBA175 (B) antibodies, and visualized by SR-SIM. The *xz* plane and *yz* plane are shown at the top and right, respectively, for panel B. The crosshairs indicate one of the limited sites of colocalization. Bars, 2 µm.

Previous reports have identified specialized apical organelles, including the PfSUB1-containing exoneme (18) and the PfROM1-containing mononeme (35), that do not overlap with classic micronemes, rhoptries, and dense granules. In order to further identify the localization of PfCDPK5, we costained parasites with markers directed against proteins in the rhoptries and micronemes. Results from SR-SIM showed that PfCDPK5 did not consistently colocalize with the rhoptry neck marker PfRON4 (36) or the exoneme marker PfSUB1 (Fig. S2B). However, we note that there are limited areas of overlap between PfCDPK5 and all of the tested apical organelle markers, mostly observed when PfCDPK5 had strong expression levels. Given the strong colocalization with PfAMA1-containing micronemes, we expected to see strong colocalization with PfEBA175, an invasion ligand residing in the micronemes (37–39). Interestingly, we found that PfCDPK5 did not colocalize as strongly with PfEBA175 as it did with PfAMA1 (Fig. 2A). The limited regions of colocalization seen with high levels of PfCDPK5 expression largely correspond to the diffuse apical staining of PfCDPK5 and not the more intense punctate regions of PfCDPK5 staining. To confirm this result, we costained parasites with antibodies against PfAMA1 and PfEBA175. As has been hypothesized before by epifluorescence and immunoelectron microscopy (40), we found that at superresolution, these two proteins show limited regions of colocalization and largely define different subsets of micronemes (Fig. 2B). Thus, when visualized at superresolution, we found that PfCDPK5 and PfAMA1 colocalize but that PfAMA1 and PfEBA175 appeared in different subsets of micronemes. These results provide further evidence for the existence of subsets of micronemes in *P. falciparum*, potentially with different functions.

### PfCDPK5-deficient parasites do not release micronemes.

Studies from both *Plasmodium* spp. and *T. gondii* have demonstrated a role for calcium-dependent protein kinases in apical organelle discharge. PfCDPK1 has been hypothesized to be important for microneme discharge (7). However, these experiments relied on chemical and peptide inhibition of the kinase in extracellular merozoites. In addition, inducible expression of the PfCDPK1 autoinhibitory junction domain caused a replication defect at the early schizont stage of *P. falciparum* (13). A recent characterization of a *P. falciparum* strain with an inducible knockdown of PfCDPK1 demonstrated no detected defects in parasite egress or PfAMA1 release but did demonstrate decreased invasion and release of PfEBA175 (41). The *Plasmodium berghei* ortholog of PfCDPK1 is not essential for blood-stage replication and therefore unlikely to be essential for microneme discharge (42). Recently, PfCDPK1 was successfully knocked out in *P. falciparum*, demonstrating that the protein is important but not essential for asexual growth (43). In *T. gondii*, CDPK1 (TgCDPK1) (11) and TgCDPK3 (44–46) are essential and important, respectively, for microneme secretion. Given these precedents, we evaluated the requirement of PfCDPK5 for microneme secretion.

**FIG 3:**
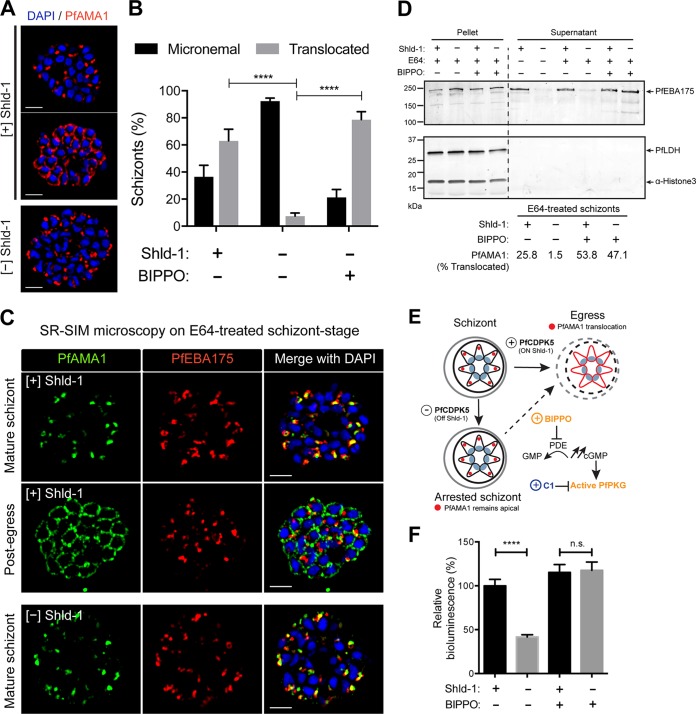
PfAMA1 translocation and egress in 3D7-PfCDPK5^DD^KnL parasites. (A) SR-SIM IFA of [+] Shld-1 schizonts or [−] Shld-1 schizonts probed with anti-PfAMA1 antibodies. Representative parasites with micronemal and translocated PfAMA1 are shown. Bars, 2 µm. (B) Quantification of each localization by wide-field IFA from [+] or [−] Shld-1 schizonts and from [−] Shld-1 schizonts treated with 2 µM BIPPO shown on the graph. Values are means plus standard deviations (SD) (error bars). Three biological replicates and 100 schizonts were counted per replicate. Values that were significantly different (*P* < 0.0001) by one-way analysis of variance (ANOVA) are indicated by bars and four asterisks. (C) SR-SIM IFAs performed on [+] Shld-1 and [−] Shld-1 3D7-PfCDPK5^DD^KnL schizonts demonstrating that the localization of PfEBA175 was not different in pre- and post-“egress trigger” [+] Shld-1 schizonts or in [−] Shld-1 schizonts. (D) Immunoblot analysis of PfEBA175 processing. [+] and [−] Shld-1 schizonts were treated with (+) or without (−) BIPPO. Equivalent amounts of schizont lysate and supernatant were subjected to immunoblot analysis with anti-PfEBA175, anti-PfLDH (loading control), and anti-H3 (α-Histone 3) (additional loading control) antibodies. In one set of supernatant samples, the schizonts were allowed to egress naturally without E64. The percentage of schizonts with translocated PfAMA1 is displayed below the immunoblot (similar to panel B, 100 schizonts counted per condition). (E) Schematic showing the effects of Shld-1, BIPPO, and compound 1 (C1) on PfAMA1 translocation. PDE, phosphodiesterase. (F) Quantification of bioluminescence in supernatant from [+] or [−] Shld-1 parasites at 50 h.p.i. with or without BIPPO treatment 90 min prior to measurement. Three biological replicates each done with technical triplicates were performed. Values are means ± SD (error bars). Values that are significantly different (*P* < 0.0001) by one-way ANOVA are indicated by a bar and four asterisks. Values that are not significantly different by one-way ANOVA (n.s.) are also indicated.

As noted above, PfAMA1 translocates from micronemes to the plasma membrane of daughter merozoites during normal parasite egress, and these “post-egress” merozoites can be obtained by inhibiting red blood cell plasma membrane rupture with E64 treatment (28, 47). Synchronized 3D7-PfCDPK5^DD^KnL ring-stage parasites were maintained in the presence and absence of Shld-1 until 46 h.p.i., E64 was added, and cultures were incubated for an additional 6 h. Harvested schizonts were evaluated for the localization of PfAMA1 (Fig. 3A). In schizonts grown in the presence of Shld-1 ([+] Shld-1 schizonts), 37% ± 8% had micronemal PfAMA1, and 63% ± 8% showed plasma membrane localization. In sharp contrast, 92% ± 2% of the schizonts grown in the absence of Shld-1 ([−] Shld-1 schizonts) had micronemal PfAMA1, and only 8% ± 2% demonstrated plasma membrane localization (Fig. 3B). It is important to note that the 3D7-PfCDPK5^DD^KnL parasites allow knockdown but do not completely knock out PfCDPK5. Thus, we find rare [−] Shld-1 schizonts with plasma membrane-localized PfAMA1. These [−] Shld-1 schizonts with translocated PfAMA1 may be from residual low levels of PfCDPK5 or from a less utilized PfCDPK5-independent pathway. Nonetheless, these results show a bona fide defect in microneme release in PfCDPK5-deficient parasites.

To simultaneously evaluate the status of the PfAMA1- and PfEBA175-containing subsets of micronemes, we costained [+] and [−] Shld-1 schizonts for the two markers (Fig. 3C). In both [+] and [−] Shld-1 schizonts, when PfAMA1 was apical, we found that PfEBA175 was also apical. Interestingly, in the “post-egress trigger” [+] Shld-1 schizonts, we found that while PfAMA1 had been translocated, on the merozoite surface, PfEBA175 apparently remained apical. When released, PfEBA175-containing micronemes expose the ectodomain of PfEBA175 onto the surface of the apical end of the merozoite. The exposed PfEBA175 is processed by the rhomboid-like protease PfROM4 on the surface of the merozoite, shedding the ectodomain into the culture supernatant (48, 49). Shedding of processed forms of invasion ligands is detected in the supernatant from E64-treated schizonts (8). Therefore, to directly evaluate whether PfEBA175-containing micronemes had been released, we measured the discharge of the processed protein into the supernatant. With E64-treated schizonts, we found that processed PfEBA175 was readily detected in the supernatant of [+] Shld-1 schizonts but was largely absent from the supernatant of [−] Shld-1 schizonts (Fig. 3D). Thus, although the sensitivity of superresolution microscopy was insufficient to detect discharge of PfEBA175-containing micronemes, their discharge was detected by measurement of the release of the processed form of PfEBA175. Together, these results demonstrate that the signaling pathways controlling the release of PfAMA1-containing and PfEBA175-containing micronemes are regulated by PfCDPK5.

### Pharmacologically enhanced PKG activity allows for microneme discharge in PfCDPK5-deficient schizonts.

PKG signaling is known to be essential for both exoneme and microneme release (8). PKG enzymatic activity is amenable to small-molecule inhibition and activation in live parasites (schematic in Fig. 3E). The kinase itself is directly and reversibly blocked by treatment with 4-[2-(4-fluorophenyl)-5-(1-methylpiperidine-4-yl)-1H-pyrrol-3-yl]pyridine (compound 1 [C1]) (50). In contrast, PKG activity is enhanced by increasing cyclic GMP levels with the specific phosphodiesterase inhibitor 5-benzyl-3-isopropyl-1H-pyrazolo[4,3-d]pyrimidin-7(6H)-one (BIPPO) (51). We examined whether BIPPO-enhanced PKG activity could overcome the PfCDPK5-mediated defect in PfAMA1 translocation. Following treatment with 2 μM BIPPO, PfAMA1 translocation was observed in 78% ± 6% of [−] Shld-1 schizonts (Fig. 3B). Similar to our findings with PfAMA1, processed PfEBA175 was robustly detected in the supernatants of both [+] and [−] Shld-1 schizonts following BIPPO treatment (Fig. 3D).

Utilizing our bioluminescent transgenic reporter parasites (Fig. 1B), we found that [−] Shld-1 parasites, even with a >90% block as schizonts, still release some KnL into the supernatant. We note that the amount of bioluminescence in the supernatant of [−] Shld-1 parasites was not further decreased when these parasites were treated with a mixture of protease inhibitors (including serine, cysteine, and metalloprotease inhibitors) at 44 h.p.i. (Fig. S3A). Thus, it is likely that the PfCDPK5-deficient arrested schizonts have partial permeabilization of their surrounding PVM and RBC plasma membrane that is not dependent on ongoing protease activity. This finding is consistent with previous reports that the PVM and RBC plasma membrane of segmented schizonts are partially permeable to small proteins even in the presence of E64 (8, 28). Nonetheless, the significant difference in the quantity of bioluminescence in the supernatant between [+] and [−] Shld-1 schizonts allowed a quantitative measure of membrane permeability as a surrogate for relative egress efficiency. To test whether BIPPO activation of PKG induced PfCDPK5-deficient parasites to egress, we measured the bioluminescence in the supernatant of treated parasites. As expected, the amount of bioluminescent reporter released into the supernatant from [+] and [−] Shld-1 parasites was equivalent following BIPPO treatment (Fig. 3F). Pretreatment of [−] Shld-1 parasites with C1 prior to BIPPO prevented this release (Fig. S3A). For additional confirmation of this result, we treated PfCDPK5-deficient parasites with zaprinast, a structurally distinct phosphodiesterase inhibitor that increases cGMP levels and activates PfPKG, and obtained similar release of bioluminescent activity into the supernatant (Fig. S3B). This result demonstrates that enhanced PKG activity induces microneme secretion and secondarily mediates full permeabilization of the PVM and RBC plasma membrane.

**FIG 4:**
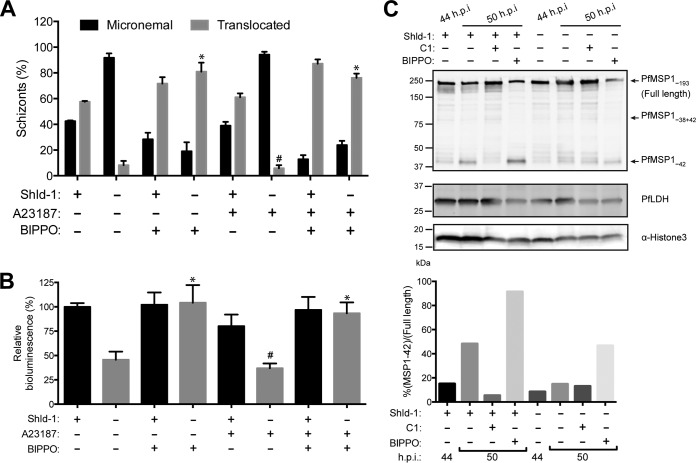
Calcium and cGMP signaling pathways in 3D7-PfCDPK5^DD^KnL parasites. (A) PfAMA1 translocation was visualized by wide-field IFA from [+] Shld-1 or [−] Shld-1 parasites treated with 1 µM A23187 and/or 2 µM BIPPO as indicated. Three biological replicates were performed, and 100 schizonts counted per replicate. Values are means plus SD (error bars). The percentage translocated from the small-molecule-treated [−] Shld1 samples was compared to the value for the untreated [−] Shld1 condition by one-way ANOVA. Values that were significantly different (*P* < 0.001) by one-way ANOVA are indicated by an asterisk. Values that were not significantly different (#) by one-way ANOVA are also indicated. (B) Bioluminescence activity released in culture supernatants from the same conditions as in panel A (three biological replicates each done with technical triplicates; mean ± SD). Each of the [−] Shld1 with small-molecule treatments was compared to the untreated [−] Shld1 condition by one-way ANOVA (*, *P* < 0.001; #, not significantly different). (C) 44 h.p.i. schizonts from [+] or [−] Shld-1 parasites were isolated by magnetic purification and lysed immediately or incubated for an 6 additional hours with the indicated compounds prior to lysis. Protein lysates were subjected to immunoblot analysis with anti-PfMSP1_42_, anti-PfLDH (loading control), and anti-H3 (additional loading control) antibodies. Full-length, partially processed, and fully processed PfMSP1_42_ are labeled. The quantitative ratio of fully processed PfMSP1_42_ relative to the unprocessed form was calculated by volumetric measurement of fluorescence intensity with the Li-Cor Odyssey CLx system.

10.1128/mBio.00130-18.4 FIG S3  Measurements of bioluminescence release and PfSERA5 processing. (A) Bioluminescence activity released by [+] Shld-1 or [−] Shld-1 3D7-PfCDPK5^DD^KnL parasites treated with the indicated compounds (*n* = 3; mean ± SD). The protease inhibitor (PI) cocktail (SIGMAFAST tablet) was used at the manufacturer’s recommended concentration. (B) The effect of treating [−] Shld-1 3D7-PfCDPK5^DD^KnL schizonts with 100 µM zaprinast was similar to that of the 2 µM BIPPO treatment (*n* = 3; mean ± SD). (C) Analysis of PfSERA5 processing. 44 h.p.i. schizonts from [+] or [−] Shld-1 parasites were isolated by magnetic purification and lysed immediately or incubated for 6 additional hours with the indicated compounds prior to lysis. Protein lysates were subjected to immunoblot analysis with anti-PfSERA5 (anti-PfLDH and anti-H3 loading controls shown in Fig. 4C). Full-length, partially processed, and fully processed PfSERA5 are labeled. (D) Quantitative ratio of fully processed PfSERA5 (p50 plus p56) relative to the unprocessed form was calculated by volumetric measurement of fluorescence intensity with the Li-Cor Odyssey CLx system. Download FIG S3, TIF file, 23 MB. Copyright © 2018 Absalon et al. 2018 Absalon et al. This content is distributed under the terms of the Creative Commons Attribution 4.0 International license.

PKG activity has been linked to downstream calcium release in both *Plasmodium* species and *T. gondii* (52–54). It is formally possible that PKG-induced increases in cytoplasmic calcium may be sufficient to activate a residual amount of PfCDPK5 in [−] Shld-1 parasites. To test whether the BIPPO-induced microneme discharge in PfCDPK5-deficient parasites was entirely dependent on calcium release, we evaluated PfAMA1 localization (Fig. 4A) and release of the KnL reporter in the supernatant (Fig. 4B) in three biological replicates of [+] and [−] Shld-1 3D7-PfCDPK5^DD^KnL schizonts treated with either BIPPO, a calcium ionophore (A23187), or both. In PfCDPK5-deficient schizonts, A23187 treatment did not induce PfAMA1 translocation, suggesting that calcium release by itself was not sufficient for microneme discharge. In [+] Shld-1 control parasites, A23187 treatment did not inhibit PfAMA1 translocation, showing that the elevated level of calcium was not toxic or inhibitory for microneme discharge. This conclusion is further supported by the observation that the combination of A23187 and BIPPO in the parasites had the same result as that for BIPPO alone in both [+] and [−] Shld-1 schizonts. Therefore, the BIPPO-induced microneme release and resulting egress in PfCDPK5-deficient schizonts are not solely due to a secondary PfPKG-induced calcium release.

### Egress protease cascade is slowed in PfCDPK5-deficient parasites.

Previously, we observed that the egress protease cascade was similarly initiated in both [+] and [−] Shld-1 schizonts (15). However, that observation was done using a single time point. Thus, we evaluated the extent to which the protease cascade in PfCDPK5-deficient parasites progressed with time—as has been demonstrated for wild-type parasites (8). For this set of experiments, we chose to use the previously published 3D7-PfCDPK5-DD parasites without an epitope tag (15). This strain was selected, because it has the strongest replication block of our existing strains. To evaluate the protease cascade, we focused on PfSUB1 processing of the *P*. *falciparum* merozoite surface protein-1 (PfMSP1), as this protein is known to be important for parasite egress (20). Following exoneme release, PfSUB1 proteolytically processes the ~200-kDa PfMSP1 precursor and releases a 42-kDa product, PfMSP1_42_. We isolated 44 h.p.i. schizonts from parallel cultures that had been maintained with and without Shld-1. These isolated schizonts were sampled immediately and then allowed to incubate for an additional 6 h in the presence of E64. As seen previously, PfMSP1_42_ was already detected at the starting time point in both [+] and [−] Shld-1 samples (Fig. 4C). In the [+] Shld-1 schizonts, the amount of fully processed PfMSP1_42_ relative to the unprocessed precursor increased from <20% at 44 h.p.i. to >80% 6 h later. In contrast, in the [−] Shld-1 parasites, the relative amount of fully processed PfMSP1_42_ remained <20% at the later time point.

To evaluate whether exoneme release was completed at the initial time point, [+] Shld-1 parasites were treated with C1 to prevent any further PKG activity, and samples were collected at the later time point. Compared to the untreated [+] Shld-1 schizonts and the starting samples, the relative amount of fully processed PfMSP1_42_ was lower, indicating that protease processing was incomplete at the starting time point and that processing required PfPKG activity. As expected, treatment of [−] Shld-1 schizonts with C1 did not change the relative amount of the fully processed PfMSP1_42_. However, when the [−] Shld-1 schizonts were treated with BIPPO, the relative amount of fully processed PfMSP1_42_ increased to >40% (Fig. 4C). These results demonstrate a previously unrecognized finding—that protease processing is incomplete in [−] Shld-1 schizonts and that full processing is increased when PfPKG activity is chemically enhanced by BIPPO. We evaluated PfSERA5, another substrate of PfSUB1 processing (18), in parallel (Fig. S3C). While the processing differences are smaller, we observed an increase in the relative amount of the mature (p50/p56) forms of PfSERA5 in the [+] Shld-1 schizonts after 6 h and did not observe the same increase in the [−] Shld-1 schizonts (Fig. S3D). As was seen with PfMSP1, the amount of mature PfSERA5 increased in both [+] and [−] Shld-1 schizonts following BIPPO treatment.

### Physical release of merozoites overcomes microneme release defect.

**FIG 5:**
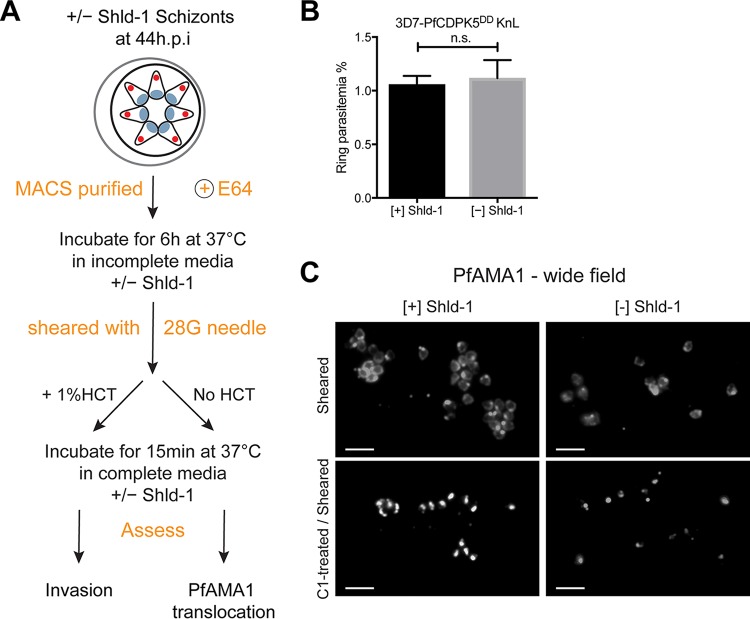
Physically released merozoites are invasive. (A) Schematic of the method to generate viable free merozoites efficiently from [+] Shld-1 and [−] Shld-1 schizonts. 28G, 28-gauge; HCT, hematocrit. (B) Ring parasitemia from physically released [+] or [−] Shld-1 schizonts. The ring parasitemia was not different between the [+] and [−] Shld-1 conditions (*n* = 3; mean ± SD; no significant difference by Student’s *t* test). (C) Wide-field IFA of PfAMA1 localization before and after shearing from [+] and [−] Shld-1 parasites. Treatment of schizonts with 2.5 µM C1 prior to shearing prevents PfAMA1 translocation.

A recently developed procedure to obtain significant quantities of viable merozoites has allowed for direct studies of mechanically released parasites (27, 55, 56). We have previously shown that PfCDPK5-deficient merozoites were capable of reinvasion following physical disruption of the blocked schizonts (15). However, the invasion obtained by this method was inefficient compared to [+] Shld-1 schizonts. We therefore modified the published “viable merozoite isolation” (27) procedure to directly evaluate the status of PfCDPK5-deficient merozoites (Fig. 5A). Applying this technique, we found that invasion efficiency of physically released merozoites from [+] and [−] Shld-1 schizonts was similar and more robust than previously reported (Fig. 5B). We evaluated PfAMA1 localization in the schizont prior to shearing and in the merozoites following physical release. Once again, the [+] Shld-1 schizonts had populations of both micronemal and translocated PfAMA1, while [−] Shld-1 schizonts had almost exclusively micronemal localization. Following shearing, translocated PfAMA1 was readily detected on physically released merozoites from both [+] and [−] Shld-1 schizonts (Fig. 5C). We repeated the shearing in the presence of C1 to evaluate whether PfAMA1 translocation induced by physical release required PfPKG activity. By IFA, PfAMA1 remained micronemal in released merozoites from C1-treated [+] or [−] Shld-1 schizonts (Fig. 5C; shown with nuclear counterstain in Fig. S4). Thus, the process of physical disruption and/or exposure of PfCDPK5-deficient merozoites to the extracellular milieu was sufficient to facilitate microneme release, as measured by PfAMA1 translocation, and this release required PfPKG activity. By preventing PfAMA1 translocation, inhibition of PfPKG prevented reinvasion and new ring formation. The mechanism responsible for the enhanced PfPKG activity in the sheared merozoites remains unknown. However, these findings convincingly demonstrate that a primary role of PfCDPK5 is to trigger microneme release, allowing parasite egress.

10.1128/mBio.00130-18.5 FIG S4  Wide-field IFA of physically released merozoites (same fields as shown in Fig. 5C) with PfAMA1 (red) and DAPI counterstaining of nuclei (blue). The bottom panels show that the PfAMA1 translocation observed in physically released merozoites is inhibited by C1 treatment. Download FIG S4, TIF file, 17.7 MB. Copyright © 2018 Absalon et al. 2018 Absalon et al. This content is distributed under the terms of the Creative Commons Attribution 4.0 International license.

## DISCUSSION

PfCDPK5 is essential for *P. falciparum* egress out of an infected RBC. It localizes to the apical ends of newly forming daughter merozoites, and in mature, segmented schizonts, PfCDPK5 colocalizes with PfAMA1; later, PfCDPK5 has a diffuse apical staining that overlaps with several apical markers. PfCDPK5 is present in the membrane-associated fraction following sodium carbonate extraction, and this localization may be due to potential palmitoylation sites within the protein (15). However, the trafficking determinants for PfCDPK5 remain unknown. By superresolution microscopy, we demonstrate multiple nonoverlapping subsets of micronemes. This result has been previously noted for PfSUB1 (the “exoneme”) and PfROM4 (the “mononeme”) (18, 35). However, we suggest that the current list of apical organelles should include additional subsets of egress-specific micronemes. We hypothesize that a subset of micronemes is triggered for release by cooperative activation of PfPKG by PfCDPK5. This defect is most convincingly demonstrated for the PfAMA1-containing micronemes. The complete identities of which parasite proteins reside in these micronemes remain unknown but likely include proteases, perforin-like proteins, and potentially other unknown proteins. We hypothesize that PfCDPK5 interacts with and likely phosphorylates proteins that are associated with apical organelles to transmit a “release” signal.

We note that parasites physically released following PfPKG-blockade by compound 1 (C1) do not translocate PfAMA1 to the merozoite surface (Fig. 5C; also see Fig. S4 in the supplemental material) and are, therefore, not invasive. In contrast, PfCDPK5-deficient parasites can reinvade new RBCs following physical release. Thus, blocking parasites prior to exoneme release prevents both egress and invasion. Our current findings demonstrate an essential role of PfCDPK5 for a downstream release of egress-mediating micronemes, and we show that chemically enhanced PfPKG activation can overcome the egress block in PfCDPK5-deficient parasites.

**FIG 6:**
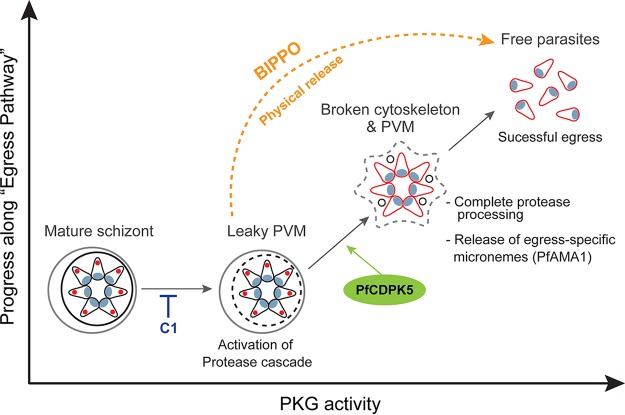
Model for cooperativity between PfCDPK5 and PfPKG. The progress along the parasite egress pathway is promoted by activation of PfPKG. At low levels of PfPKG activation, the protease cascade and calcium signaling pathway are initiated. PfCDPK5 activation, together with PfPKG, leads to further progression of the protease cascade and triggered release of micronemes required for parasite egress (detected by PfAMA1 translocation, shown in red). Under physiological conditions, this step requires both PfCDPK5 and PfPKG. The discharge of micronemes completes the egress process with the release of invasive merozoites. The requirement for PfCDPK5 can be bypassed by supraphysiological activation of PfPKG by either BIPPO or physical disruption. PVM, parasitophorous vacuolar membrane.

We, therefore, propose a model of egress wherein PfCDPK5 cooperates with PfPKG activation (Fig. 6). In this model, a basal level of PfPKG activation allows some release of exonemes and partial activation of the protease cascade. This initial step is independent of PfCDPK5. In a later step, PfCDPK5 cooperates with PfPKG to facilitate PfAMA1 translocation and discharge of micronemes required for egress. This second step, requiring PfPKG and PfCDPK5, induces full activation of the protease cascade and allows parasite egress. Finally, supraphysiological activation of PfPKG by BIPPO treatment can bypass the requirement for PfCDPK5. One alternative to the model is that PfCDPK5 activity does not affect exoneme secretion directly but rather functions to increase the efficiency and/or completeness of protease processing after exoneme release.

Our methods to obtain free PfCDPK5-deficient merozoites require either BIPPO (or zaprinast) treatment or physical disruption. These methods facilitate PfAMA1 translocation and likely the release of other egress-specific factors. We cannot formally rule out a role for PfCDPK5 in the invasion process that was not also “overcome” by these treatments. Nonetheless, PfCDPK5 is the only calcium-dependent protein kinase in *Plasmodium* spp. that has a direct role in the release of micronemes required for parasite egress.

Synergy between calcium-dependent kinases and PKG has been suggested by previous studies with both *T. gondii* and *Plasmodium* parasites (57–59). Our current results provide strong genetic evidence for this hypothesis. Brochet and colleagues evaluated the relationship between *P*. *berghei* PKG (PbPKG) and calcium signaling, providing a molecular pathway for connection of the two pathways (52). Alam and colleagues demonstrated that PfCDPK1 was a likely substrate of PfPKG and that phosphorylated PfCDPK1 localized to the apical area of daughter merozoites (60). Bansal and colleagues demonstrated that increased PfPKG activity was able to compensate for a less active PfCDPK1 (57). Similarly, Moon and colleagues demonstrated that increased PbPKG signaling was able to overcome a defect in gliding motility observed in PbCDPK3-deficient ookinetes (59). Our current study adds to the complexity of the egress signaling pathway by demonstrating a cooperative relationship between PfCDPK5 and PfPKG activity.

In summary, we have demonstrated a functional explanation for the egress block observed in PfCDPK5-deficient parasites. In the absence of normal PfCDPK5 activity, physiologic activation of the PfPKG pathway is insufficient to release a subset of micronemes that are essential for parasite egress. Thus, the molecular mechanism for the egress block observed in PfCDPK5-deficient schizonts is secondary to the failure to discharge micronemes that are required for egress.

## MATERIALS AND METHODS

### Small molecules and antibodies.

Synthesis of 5-benzyl-3-isopropyl-1H-pyrazolo[4,3-d]pyrimidin-7(6H)-one (BIPPO) was adapted from published methods (51, 61) and dissolved at 10 mM in dimethyl sulfoxide (DMSO). See Text S1 in the supplemental material for details. Compound 1 (C1) {4-[2-(4-fluorophenyl)-5-(1-methylpiperidine-4-yl)-1H-pyrrol-3-yl]pyridine} (50 mM), E64 (10 mM), A23187 (50 mM), and zaprinast (10 mg/ml) were dissolved in DMSO. Commercially available antibodies were obtained from Roche Applied Science (rat antihemagglutinin [anti-HA] [3F10]), Life Technologies (mouse anti-HA [clone 2-2.2.14]), and Abcam (rabbit anti-H3). Other antibodies were generously provided by Michael Makler at Flow Inc. (mouse anti-*P*. *falciparum* lactate dehydrogenase [anti-PfLDH]), Julian Rayner at the Wellcome Trust Sanger Institute (rabbit anti-PfGAP45), Robin Anders at The Walter & Eliza Hall Institute of Medical Research (mouse anti-PfAMA1 [clone 1FG]), Jean-Francois Dubremetz at Universitè Montpellier (mouse anti-PfSERA5 [clone 24C6.1F1]), Alan Cowman, Jenny Thompson, and Kaye Wycherley at The Walter & Eliza Hall Institute of Medical Research (rabbit anti-PfEBA175 and mouse anti-PfRON4), Michael Blackman at the London School of Hygiene and Tropical Medicine and the Francis Crick Institute (rabbit anti-PfSUB1), Carole Long at NIAID, NIH (rabbit anti-PfMSP1_42_), and Kim Lee Sim via BEI Resources, NIAID, NIH (mouse anti-PfEBA175 [clone R218]).

10.1128/mBio.00130-18.1 TEXT S1  Supplemental methods. Download TEXT S1, DOCX file, 1.5 MB. Copyright © 2018 Absalon et al. 2018 Absalon et al. This content is distributed under the terms of the Creative Commons Attribution 4.0 International license.

### Parasite culture and transfection.

The 3D7 strain (Walter & Eliza Hall Institute) and transgenic derivatives were cultured in human red blood cells (RBCs) in RPMI 1640 supplemented with 0.5% Albumax II, 50 mg/liter hypoxanthine, 0.21% sodium bicarbonate, and 25 mM HEPES as previously described (62). To generate the 3D7-PfCDPK5^DD^ strain, sorbitol-synchronized ring-stage parasites were electroporated with 100 μg of plasmid DNA of the single-crossover plasmid (see Text S1 for details). Following transfection, parasites were maintained with 250 nM Shield-1 (Shld-1), and stable single-crossover parasites were selected by cycling on and off WR99210 (Jacobus Pharmaceutical Company). Individual transgenic clones were obtained by limiting dilutions. To generate the 3D7-PfCDPK5^DD^KnL parasites with the KnL reporter, a clone was transfected with pJDD250 that expresses the Bxb1 integrase (23) and the KnL reporter and selected on blasticidin (while maintaining WR99210 and Shld-1).

### Parasite phenotypic assays.

For replication curves, ring-stage parasites were washed to remove Shld-1, replated in the presence or absence of 0.25 mM Shld-1 at 0.4% parasitemia and 1% hematocrit, and parasitemia was monitored by flow cytometry using SYBR green I staining. For measurements of bioluminescence throughout the asexual development cycle, 100 µl of resuspended culture was removed from ring, trophozoite, schizont, and reinvaded rings and centrifuged at 14,000 rpm in a microcentrifuge. The supernatant was transferred to a fresh tube, and 20 µl was mixed 1:1 with nano-Glo buffer/substrate (Promega). The pelleted cells were resuspended in 100 µl of phosphate-buffered saline (PBS), and then 20 µl of the PBS solution containing cells was mixed 1:1 with nano-Glo buffer/substrate. Light output was measured on a SpectraMax L instrument.

For all indirect immunofluorescence assays (IFAs), parasites were synchronized by Percoll purification of late-stage schizonts, followed by sorbitol synchronization of newly invaded rings 2 h later. In the following cycle, sorbitol synchronization was repeated, and the cultures were washed and replated with or without Shld-1. IFAs were performed as previously described (47) with minor modifications. Thin smears were made on glass slides, air dried, and fixed with 1% paraformaldehyde, permeabilized with 0.1% Triton 100 for 10 min, and blocked with 3% bovine serum albumin (BSA) overnight at 4°C. Primary antibodies were incubated overnight in a cold room at the dilutions indicated: anti-PfAMA1 (1:200), anti-PfEBA175 (1:500), anti-PfGAP45 (1:5000), anti-HA (1:50), anti-PfSUB1 (1:500), and anti-PfRON4 (1:200). Subsequently, cells were washed three times with PBS and incubated for 45 min with the Alexa Fluor 488 or 555 secondary antibodies (1:1,000) (Molecular Probes). After removal of unbound antibodies with three PBS washes, slides were mounted with Vectashield containing 4′,6′-diamidino-2-phenylindole (DAPI) (Vector Laboratories Inc.) with coverslips and kept at 4°C until evaluation. Wide-field images were obtained with a Nikon E800 epifluorescence microscope using a 100× (oil) objective, and images were captured using SPOT Imaging software and then processed using Adobe Photoshop. Superresolution structured illumination microscopy (SR-SIM) Z-stacks were captured using an ELYRA PS.1 microscope (Carl Zeiss Microscopy). The ELYRA microscope was used with a 100× (oil) objective and excitation wavelengths of 405, 488, and 561 nm. SIM images were collected at 100- to 200-nm *z*-axis steps, with five rotations of the structured illumination grid per channel. The resulting stacks were processed using default reconstruction parameters in ZEN 2012 Black software.

For the *P*. *falciparum* AMA1 (PfAMA1) translocation IFAs shown in Fig. 3, 10 µM E64 was added to the schizonts 46 h postinvasion (h.p.i.) and incubated for 4 h, then 2 µM BIPPO was added for an additional 2 h, and schizonts were harvested for analysis. For the bioluminescence and PfAMA1 translocation IFAs shown in Fig. 4, 10 µM E64 was added to 46 h.p.i. schizonts and incubated for 150 min, 1 µM A23187 (or nothing) was added for 90 min, then 2 µM BIPPO was added for 2 h, and schizonts/supernatants were harvested for analysis. A total of 100 schizonts were scored for micronemal or plasma membrane translocation, and bioluminescence was measured as described above. For PfEBA175 processing experiments, synchronized ring-stage parasites were washed three times to remove Shld-1, and grown at 4% hematocrit in the presence and absence of 250 nM Shld-1. At 44 h.p.i., schizonts were purified by passage through a magnetically activated cell sorting (MACS) column, spun down to a pellet, and resuspended in 100 µl of medium containing 10 µM E64 in the presence or absence of Shld-1, and plated in a 96-well plate. Heparin (50 µg/ml) was added to all wells to prevent reinvasion. BIPPO (2 μM) was added 4 h after plating, and parasites were collected 2 h after the addition of BIPPO. Samples were spun down to separate supernatant and pellet. Parasite pellets were washed once with RMPI 1640, and supernatants were spun again to clear any remaining parasites. Washed pellets and cleared supernatants were boiled in sample buffer, analyzed by immunoblotting with anti-PfEBA175 antibodies or anti-PfLDH and anti-H3 antibodies to control for loading, probed with Li-Cor secondary antibodies, and visualized on a Li-Cor Odyssey CLx system. Concurrently, samples of parasites were prepared for immunofluorescence assays by preserving 300 µl each of Shld-1 cultures grown with Shld-1 ([+] Shld-1 cultures) and Shld-1 cultures grown without Shld-1 ([−] Shld-1 cultures) prior to MACS purification. Cultures were plated along with MACS-purified schizonts, and samples were treated as described above. At the time of sample collection, cultures were spun down, and smears were made from the RBC pellet. IFA preparation was performed as described above, and PfAMA1 translocation was determined.

For PfMSP1 and PfSERA5 processing assays, 44 h.p.i. schizonts from [+] and [−] Shld-1 cultures were purified by magnetic separation, placed in medium without Albumax II with or without Shld-1. A sample of the purified schizonts were lysed immediately in sample buffer, and the remaining schizonts were incubated for an additional 6 h before harvesting. E64 (10 μM) was added to all samples at 44 h.p.i. (for C1-treated samples, 2.5 µM C1 was also added). At 48 h.p.i., 2 µM BIPPO was added to indicated samples. All treated samples were lysed at 50 h.p.i. Lysates were separated on a TGX 4 to 20% gradient cell (Bio-Rad), transferred to polyvinylidene difluoride (PVDF) by wet transfer, immunoblotted with rabbit anti-PfMSP1_42_ or mouse anti-PfSERA5 (and anti-PfLDH and anti-H3 as loading controls), probed with Li-Cor secondary antibodies, and visualized on a Li-Cor Odyssey CLx system.

For physical disruption assays, viable merozoites were performed as previously described (27, 55) with minor modifications. Late-stage schizonts were magnet purified, incubated in medium with 10 µM E64 and without Albumax II for 6 h, and sheared with 20 strokes through a 28-gauge needle. The sheared merozoites were incubated with or without fresh RBCs at 37C for 15 min. Reinvasion was determined by flow cytometry, and PfAMA1 translocation was determined by IFA.
