# Supplementary figures and images for: Calcium-Dependent Protein Kinase 5 Is Required for Release of Egress-Specific Organelles in Plasmodium falciparum
Source: mBio. 2018 Feb 27;9(1):e00130-18. doi: 10.1128/mBio.00130-18 (PMC5829822; doi:10.1128/mBio.00130-18)

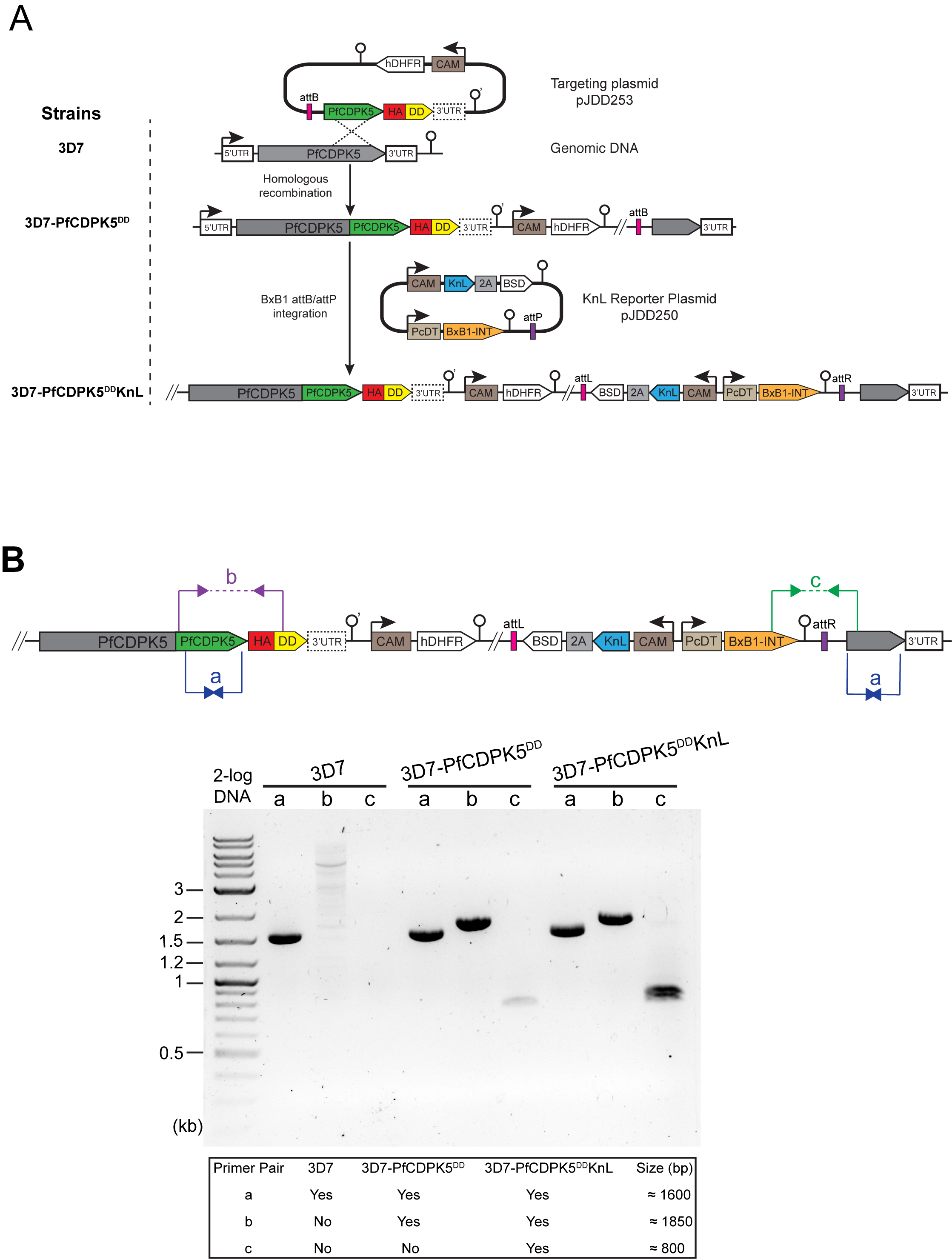

Supplement: FIG S1 [file mbo001183746sf1.tif]

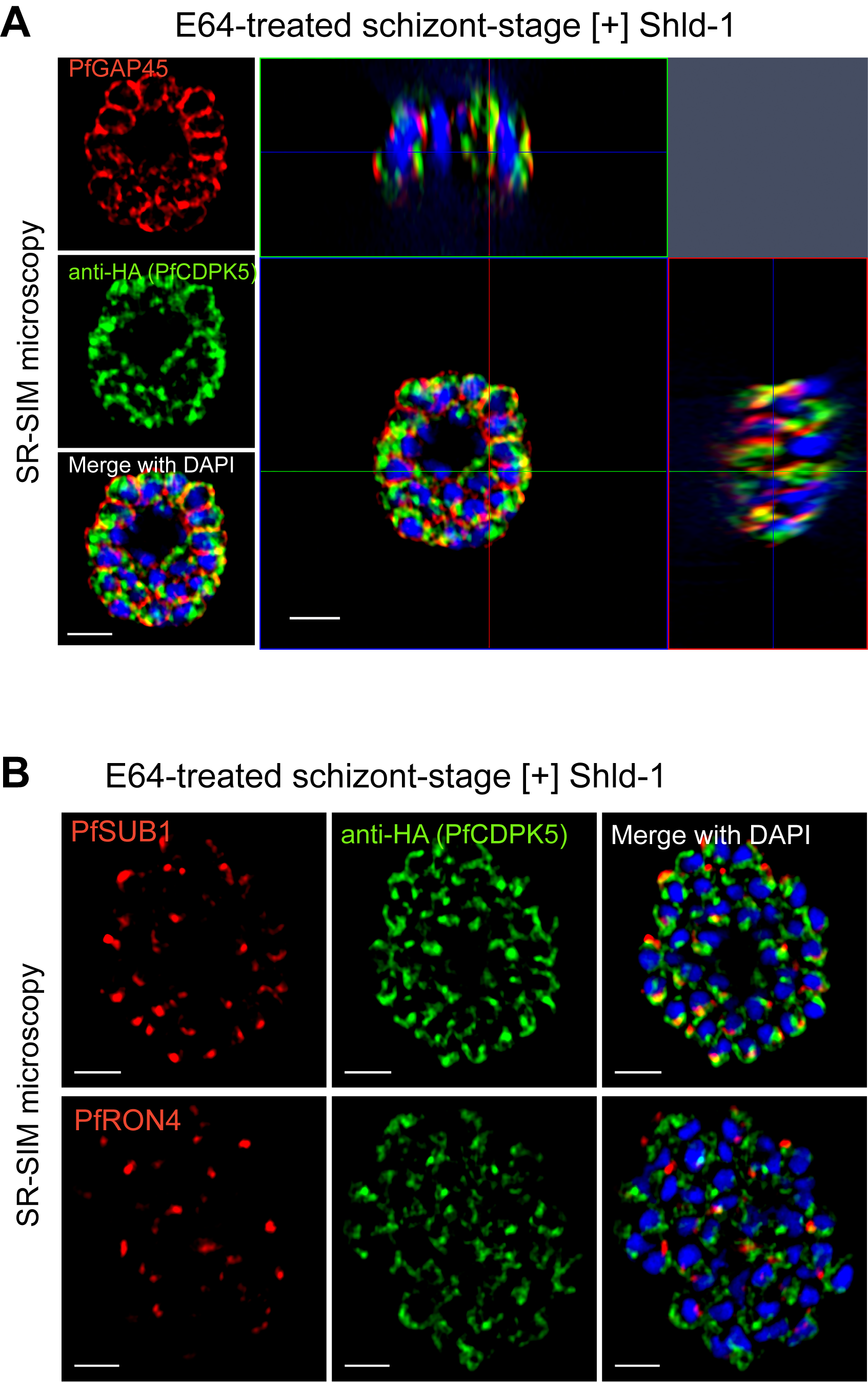

Supplement: FIG S2 [file mbo001183746sf2.tif]

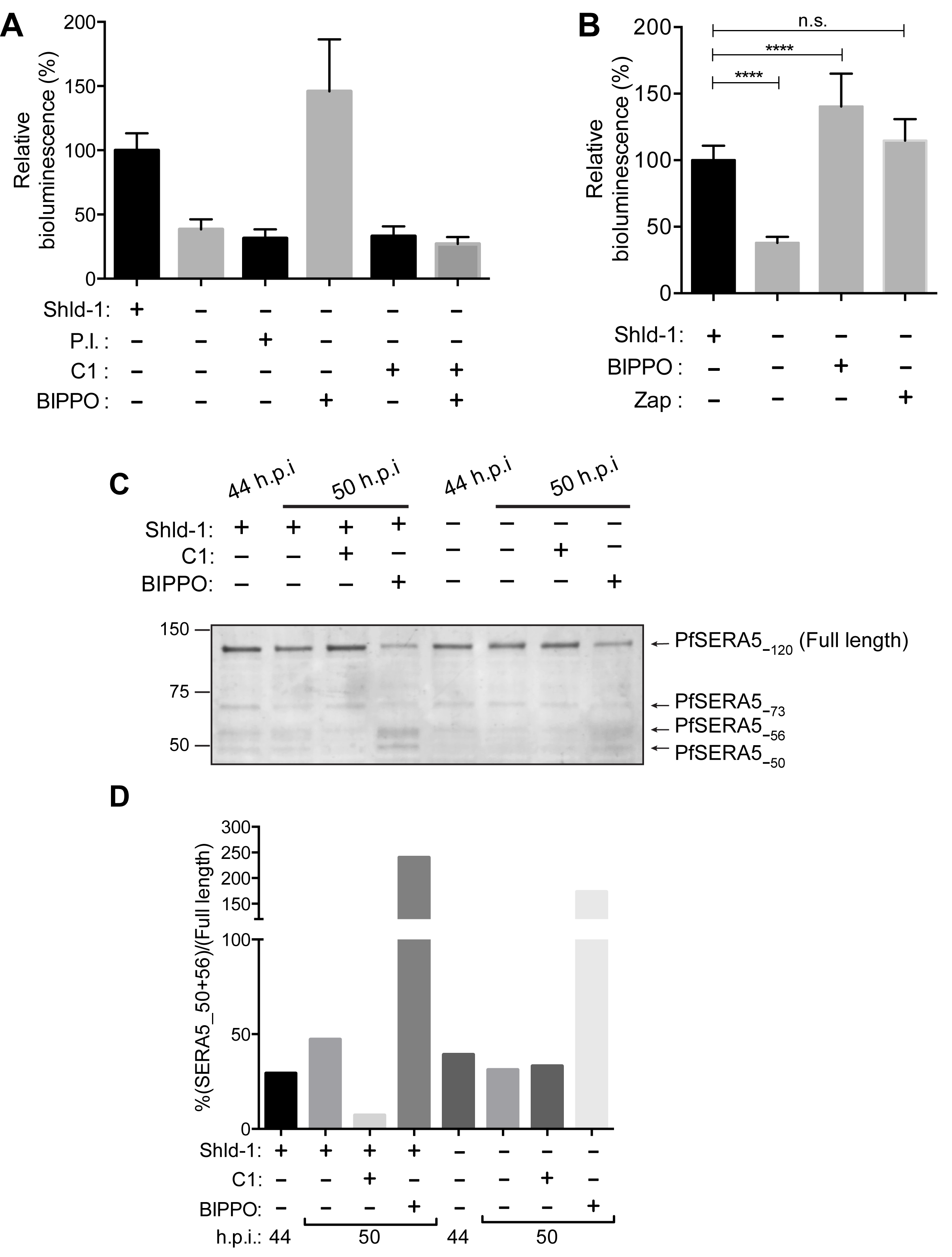

Supplement: FIG S3 [file mbo001183746sf3.tif]

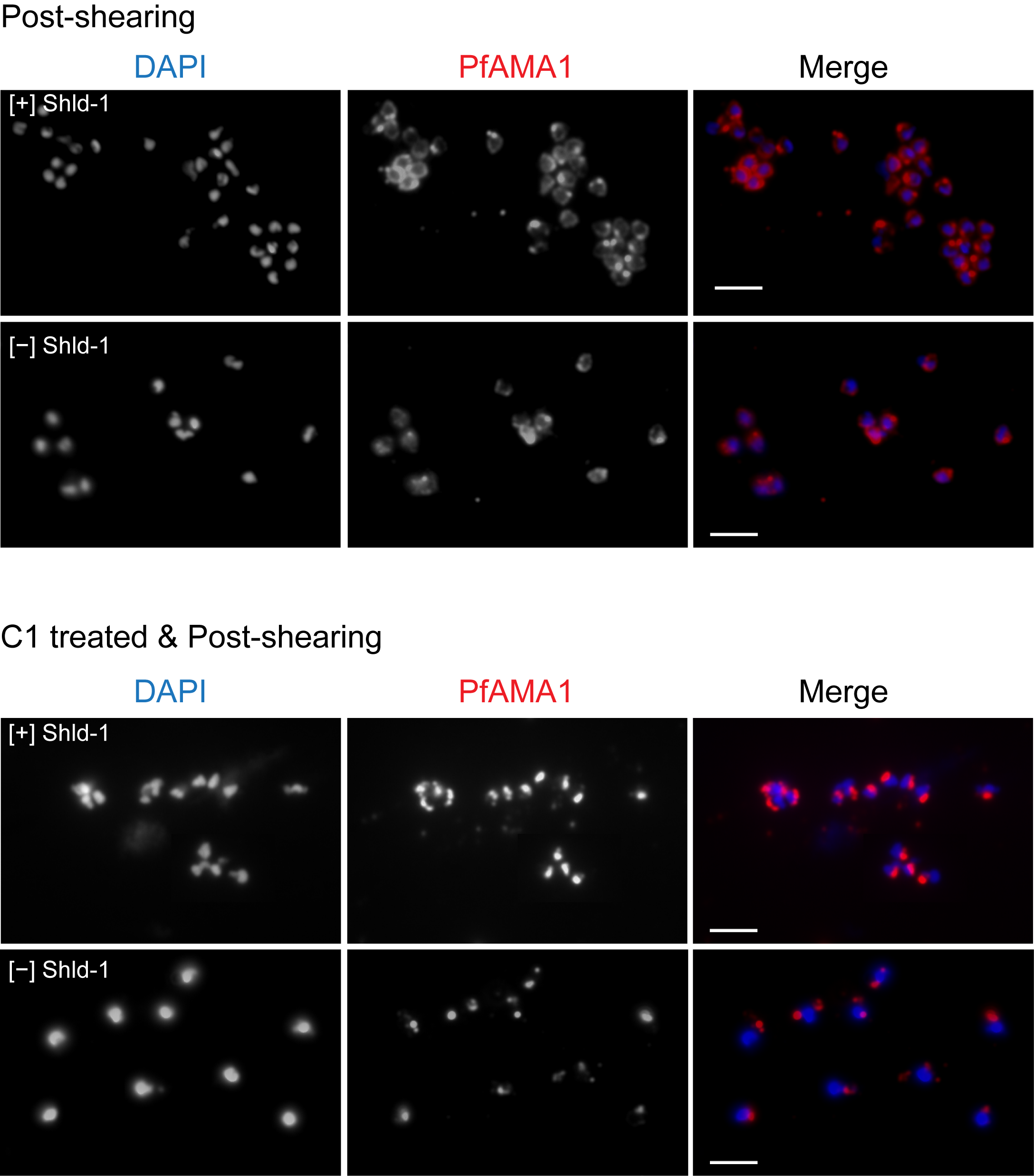

Supplement: FIG S4 [file mbo001183746sf4.tif]
