# Supplementary material for: Calcium-Dependent Protein Kinase 5 Is Required for Release of Egress-Specific Organelles in Plasmodium falciparum
Source: mBio. 2018 Feb 27;9(1):e00130-18. doi: 10.1128/mBio.00130-18 (PMC5829822; doi:10.1128/mBio.00130-18)
Supplement: TEXT S1 [file mbo001183746s1.docx]

**Supplemental Methods**

Synthesis of BIPPO.

*Synthesis of 3-isopropyl-4-(2-phenylacetamido)-1H-pyrazole-5-carboxamide intermediate*:

Phenylacetic acid (89.8 mg, 660 μmol) was dissolved in anhydrous tetrahydrofuran (6.0 mL) in a round-bottomed flask, under nitrogen. Carbonyldiimidazole (117.6 mg, 725 μmol) was added to the solution. The reaction mixture was heated to 50°C in an oil bath for 4 h. 4-Amino-3-isopropyl-1H-pyrazole-5-carboxamide (100 mg, 594 μmol, AstaTech) was added to the flask as a solution in anhydrous tetrahydrofuran (2.0 mL). The reaction was allowed to stir overnight at 50°C under nitrogen. The reaction mixture was evaporated to dryness on a rotary evaporator and the crude material was dry-loaded and purified using silica gel chromatography, eluting with 2% to 10% methanol in dichloromethane. Fractions containing product were combined, and the solvent was removed to yield the title compound as an off-white amorphous solid. ^1^H NMR (500 MHz, DMSO-*d*_6_) δ ppm 1.00 - 1.18 (m, 6 H) 2.68 - 2.93 (m, 1 H) 3.60 (s, 2 H) 7.05 - 7.38 (m, 6 H) 9.26 (br. s., 1 H) 12.85 (br. s., 1 H). LC-MS [M+H]+ 287.08 m/z.

*Synthesis of 5-benzyl-3-isopropyl-1H-pyrazolo[4,3-*d*]pyrimidin-7(6H)-one (BIPPO)*:

3-Isopropyl-4-(2-phenylacetamido)-1H-pyrazole-5-carboxamide (154 mg, 573 μmol) was dissolved in anhydrous methanol (6 mL). The solution was transferred under nitrogen to a sealed 20 mL microwave reaction vessel. A 2.5 M stock solution of sodium methoxide was prepared by dissolving sodium (92 mg) in anhydrous methanol (1.5 mL). The sodium methoxide solution (855 μL, 2.29 mmol) was then added to the microwave reaction vessel. The reaction was heated to 120°C in an oil bath overnight. The reaction was quenched by the addition of a 1 M aqueous hydrochloric acid solution (1.7 mL). The solvent was then removed on the rotary evaporator. The crude reaction mixture was dry-loaded onto silica and purified using silica gel chromatography, eluting with 0% to 5% methanol in dichloromethane. Fractions containing product were combined and the solvent was removed to yield the title compound as an off-white amorphous solid. 1H NMR (500 MHz, DMSO-*d*_6_) δ ppm 1.32 (d, *J*=7.32 Hz, 6 H) 3.17 - 3.30 (m, 1 H) 3.92 (br. s., 2 H) 7.20 - 7.25 (m, 1 H) 7.28 - 7.37 (m, 4 H) 12.27 (br. s., 1 H) 13.57 (br. s., 1 H). 1H NMR (500 MHz, METHANOL-*d*_4_) δ ppm 1.41 (d, *J*=6.35 Hz, 6 H) 3.35 - 3.52 (m, 1 H) 4.00 (br. s., 4 H) 7.20 - 7.26 (m, 1 H) 7.28 - 7.37 (m, 4 H). LC-MS [M+H]+ 269.11 m/z.

Plasmid construction. An *attB* site was added to our 3HA-DD plasmid(1) by digesting with *NotI* and *XhoI* and cloning in annealed oligonucleotides (GGCCGACGGCTTGTCGACGACGGCGGTCTCCGTCGTCAGGATCATCGCGGCCGCC and TCGAGGCGGCCGCGATGATCCTGACGACGGAGACCGCCGTCGTCGACAAGCCGTC).

To generate the PfCDPK5-3HA-DD single crossover plasmid (pJDD253), the 3’ fragment of PfCDPK5 was PCR amplified (forward primer: GCTgcggccgcGGATACGAATAGAAAAGATGGTAAAATTAAAAAG; reverse primer: TCCctcgagTTCTTTAACTCCCGTCATCATC) and cloned as *NotI* / *XhoI* fragment into a *attB*-3HA-DD plasmid. To generate the KnL reporter plasmid, the BSD-GFP cassette in pBSD-GFP-INT(2) was replaced with the KnL reporter cassette (containing the PfCAM 5’UTR – start codon - PfKAHRP(AA 1-60) – nano-luciferase coding sequence (Promega) – 2A peptide – hDHFR – stop codon – PbDT 3’ UTR) in multiple steps. The resulting plasmid, pJDD250, expresses the KnL and hDHFR proteins from a single promoter, the Bxb1 integrase protein from a second promoter, and contains a single *attP* site.

References for supplemental information

1. **Absalon S**, **Robbins JA**, **Dvorin JD**. 2016. An essential malaria protein defines the architecture of blood-stage and transmission-stage parasites. Nat Commun **7**:11449.

2. **Nkrumah LJ**, **Muhle RA**, **Moura PA**, **Ghosh P**, **Hatfull GF**, **Jacobs WR**, **Fidock DA**. 2006. Efficient site-specific integration in Plasmodium falciparum chromosomes mediated by mycobacteriophage Bxb1 integrase. Nat Meth **3**:615–621.
